# Paradoxical modulation of influenza by intranasal administration of non-replicating adenovirus particles

**DOI:** 10.1371/journal.pone.0241266

**Published:** 2020-11-12

**Authors:** De-chu Christopher Tang

**Affiliations:** VaxDome Inc., Commercialization Program, University of North Texas Health Science Center, Fort Worth, Texas, United States of America; Stanford University School of Medicine, UNITED STATES

## Abstract

Respiratory mucosal infection by airborne microbes is a common event that occurs every day. We report here that intranasal administration of non-replicating adenovirus (Ad) particles to mice could either confer rapid protection against influenza virus (IFV) challenge independent of adaptive immunity, or exacerbate influenza by triggering rapid death. The life-or-death outcome hinges on the time interval between Ad administration and IFV challenge in conjunction with specific mouse/IFV strains. Intranasal instillation of Ad particles 1–47 days prior to IFV challenge conferred rapid protection against influenza in Balb/c mice whereas exposure to Ad 39 days prior to challenge with a specific IFV strain or 1 day post-challenge with that IFV strain induced rapid death in C57BL/6 mice. Notably, consecutive administrations of Ad prior to IFV challenge conferred a synergy in triggering a potent anti-influenza state; even a detrimental Ad exposure 39 days before challenge with the deadly IFV strain was reversed to a beneficial one by subsequent Ad boosts. Results revealed an intricate relationship between infection and innate immunity that is a linchpin around which effects revolve from protective immunity to collateral damage. It is urgent to repeat the experiments with an expanded scope for characterizing the status that defines susceptibility or resistance to IFV infection and subsequently reveal the underlying mechanisms. Whether broad heterologous protective effects induced by AdE and adaptive immunity elicited by vaccination could confer synergy during mitigation of a pandemic remains to be seen.

## Significance

**Fig 1:**
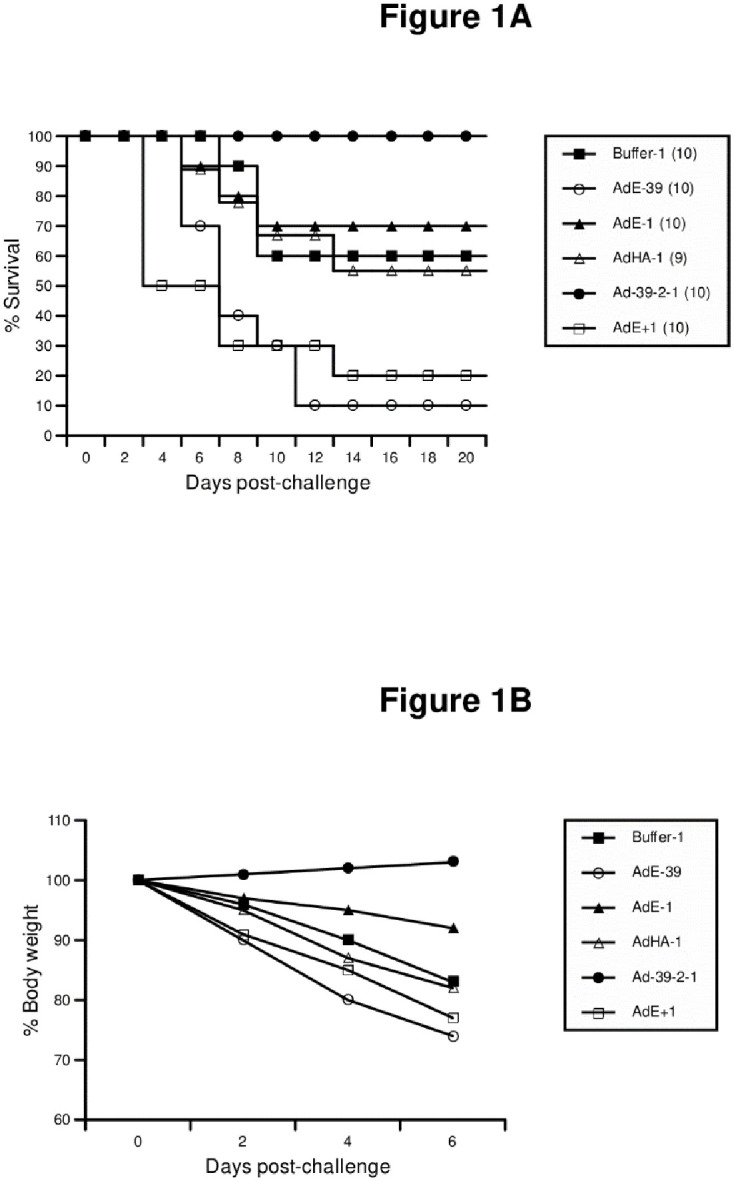
Timing of intranasal administration of nonreplicating Ad particles determines whether an influenza virus challenge is ameliorated or exacerbated in mice. Purified AdE and AdCA04HA1 particles as well as CA04 IFV particles were inoculated into young (2–3 months old) female C57BL/6 mice by intranasal instillation at different time points as described [1]. Mice were monitored daily for survival with body weights recorded every other day post-challenge. (**A**) Survival rates post-CA04 challenge; % survival was determined by taking the number of mice on Day 0 as 100%. (**B**) Body weight loss post-CA04 challenge; post-challenge body weights are presented as mean % body weight by taking the body weight of individual mice on Day 0 as 100%. Buffer-1 (negative control group inoculated with virus-free A195 buffer only), A195 buffer was instilled into the nostril one day prior to CA04 challenge; AdE-39, AdE particles were instilled into the nostril 39 days prior to CA04 challenge; AdE-1, AdE particles were instilled into the nostril 1 day prior to CA04 challenge; AdHA-1, AdCA04HA1 particles were instilled into the nostril 1 day prior to CA04 challenge; Ad39-2-1, AdE particles were instilled into the nostril 39 and 2 days prior to CA04 challenge, followed by instillation of AdCA04HA1 particles 1 day prior to CA04 challenge; AdE+1, AdE particles were instilled into the nostril 1 day after CA04 challenge; parentheses represent the number of animals in each group. AdE and AdCA04HA1 particles were instilled into the nostril at a dose of 1.6X10^8^ infectious units (ifu) in a volume of 0.05 ml per mouse; CA04 particles were instilled into the nostril at a dose of 8X10^3^ plaque-forming units (pfu) in a volume of 0.05 ml per mouse, which was equivalent to approximately 1xLD_50_ in this experiment. Statistical analysis was performed using GraphPad Prism version 8.4.3 (686) (GraphPad Software, San Diego, CA). Log-rank tests were performed for comparing Kaplan-Meier survival curves; the protection afforded in AdE-39 group (*P* = 0.0002), AdE+1 group (*P* = 0.0055), and Ad-39-2-2 group (*P* = 0.0131) reached statistical significance when compared to that of the Buffer-1 control group. Mice in the Ad-39-2-1 group significantly gained body weight over time when compared to their counterparts in the Buffer-1 control group (*P* = 0.0492; unpaired t-test). Statistical significance was set at *P*<0.05.

Although respiratory mucosal infection by airborne microbes is an everyday event, little is known about how different microbes antagonize each other in the airway. Characterization of the interactions between IFV and other mucosal viruses during co-infection or consecutive infections not only may shed light on the convoluted impact of IFV infection upon health, but also could foster the development of novel antiviral drugs that will not be impaired by drug resistance over time. We have demonstrated that intranasal instillation of AdE (nonreplicating E1/E3-defective human adenovirus serotype 5 without exogenous genes; “E” stands for “empty”) particles 1 day prior to A/California/04/2009 (CA04; mouse-adapted pandemic 2009 H1N1 swine IFV kindly provided by Dr. Robert G. Webster) challenge conferred seemingly mild protection of C57BL/6 mice against influenza whereas exposure to AdE 39 days prior to CA04 challenge or 1 day post-CA04 challenge induced rapid IFV-mediated death, more rapidly than their counterparts that were challenged with CA04 only (Fig 1A). Notably, consecutive administrations of AdE in conjunction with AdCA04HA1 (Ad vector encoding a codon-optimized CA04 HA1 gene [synthesized at GenScript]) prior to IFV challenge conferred a synergy in triggering a potent anti-influenza state (Fig 1A); even a lethal AdE exposure 39 days prior to CA04 challenge was reversed to a beneficial one by subsequent AdE/AdCA04HA1 boosts, as shown by significant mitigation of IFV-induced body-weight loss (Fig 1B). Surprisingly, AdE induced an anti-influenza state against live A/Puerto Rico/8/34 (PR8; H1N1; VR-95, ATCC) IFV in Balb/c mice in a similar but different manner [1]. Results suggested that nonreplicating AdE may be endowed with the potential for development into a broad-spectrum antiviral drug that could confer weeks-long heterologous protection of hosts against unrelated mucosal viruses in a wide variety of disease settings without impairment by drug resistance over time, because AdE most likely induces a broad-spectrum antiviral state by remodeling the airway into an Ad territory that impedes other viruses’ activities within the respiratory tract without attacking unrelated viruses directly. Since administration of AdE post-IFV challenge either conferred no protection of Balb/c mice [1] or exacerbated influenza in C57BL/6 mice (Fig 1), it is not yet feasible to mobilize AdE-mediated innate immunity toward beneficial post-exposure therapy of influenza. If our hypothesis should be correct, AdE could be developed into a post-exposure antiviral drug by mitigating the initial pro-IFV arm of innate immunity induced by IFV infection. First and foremost, we have to repeat these experiments in greater detail for determining the specific parameters that tip the scale toward pro-influenza and anti-influenza states, respectively.

## Hypothesis

It is conceivable that airborne mucosal viruses may constantly compete for territories within human and animal airways. When Ad infects the airway, it may initially program innate immunity into a pro-Ad response that impedes subsequent activities of other viruses (*e*.*g*., IFV) in the airway as suggested by our findings [1], until the pro-Ad response is later disabled by anti-Ad immunity. Likewise, a pro-IFV arm of innate immunity could be activated immediately post-IFV infection that incapacitates Ad if Ad should be introduced into the airway post-IFV infection. It would be of medical importance to characterize different arms of innate immunity activated by infection with different viruses in the airway, not only for understanding virus-host and virus-virus interactions but also for paving the way for the development of a new generation of broad-spectrum antiviral drugs that will not be impaired by drug resistance over time in a wide variety of disease settings. The urgency to develop broad-spectrum strategies against pandemics caused by unknown viruses in the future has been brought to a clear focus by the ongoing SARS-CoV-2 pandemic that is ravaging nearly the entire world with no end in sight. Although broad heterologous protective effects have been induced by a number of live vaccines, it is conceivable that they may be elicited via different mechanisms as some of the live vaccines confer beneficial protection only in neonates whereas others may protect adults [2]. Whether broad heterologous protective effects induced by different agents could be coordinated to confer synergy as a powerful tool in the public health arsenal against a pandemic remains to be determined.

## Specific aims

### Rationale

Intranasal administration of AdE particles into Balb/c mice 1, 2, 22, or 47 days prior to IFV challenge induced an anti-influenza state that conferred protection against a lethal challenge by live PR8 or CA04 IFV, whereas inoculation of AdE post-IFV challenge conferred neither mitigation nor exacerbation of influenza [1]. Intranasal administration of AdE particles into C57BL/6 mice induced a similar but different anti-influenza state (Fig 1). While AdE inoculation 1 day prior to CA04 challenge induced a seemingly mild (statistically insignificant) anti-influenza state in C57BL/6 mice, AdE administration either 39 days prior to CA04 challenge (*P* = 0.0002) or 1 day post-CA04 challenge (*P* = 0.0055) exacerbated influenza by killing C57BL/6 mice more rapidly than their counterparts challenged with CA04 only; and the lethal exposure to AdE 39 days prior to CA04 challenge could be reversed to a beneficial one by subsequent Ad boosts prior to CA04 challenge as shown by 100% survival rate (*P* = 0.0131) (Fig 1A) in conjunction with remarkable body-weight gain (*P* = 0.0492) in the face of IFV infection (Fig 1B). Similar to AdE, administration of AdCA04HA1 one day prior to CA04 challenge did not change survival rates significantly. It is unclear whether the AdE-mediated exacerbation of influenza at specific time points (Fig 1) was due to the C57BL/6 mouse strain, the CA04 IFV strain, or both. Results implied that random infection of airway by airborne microbes at varying time points may unpredictably ameliorate or exacerbate influenza or diseases caused by other airborne viruses (*e*.*g*., coronavirus; respiratory syncytial virus; *etc*.), and consecutive nasal spray of a benign, nonreplicating, bioengineered virus-derived particle such as AdE could rapidly confer protection of the host against IFV and possibly other unrelated pathogens as well by remodeling the airway into an Ad territory that impedes other viruses’ activities. Since AdE-induced anti-influenza state in Balb/c mice slowly declined away after 6 weeks post-administration [1], this novel modality may emerge as a complement to vaccination by conferring broad protection against unrelated pathogens before vaccination-elicited adaptive immunity against a specific pathogen is protective enough [3]. Notably, the safety profile and potency of an Ad-vectored nasal influenza vaccine has been demonstrated in human subjects during a human Phase I clinical trial [4]; it is thus conceivable that there should be no major safety concern for the AdE-mediated therapy option to enter human clinical trials after animal studies pan out. In these studies, we will specifically determine the parameters that tip the scale toward pro-influenza and anti-influenza states, respectively.

### Specific aim #1: Determination of the parameters that tip the scale toward an anti-influenza state or exacerbation of influenza

In these studies, we will determine whether exacerbation of influenza by AdE particles is due to the mouse strain, the CA04 IFV strain, or both. AdE particles will be produced, purified, and instilled intranasally into Balb/c and C57BL/6 mice, respectively, at different time points. As shown in Table 1, mice will be challenged by intranasal administration of live PR8 or CA04 IFV as described [1].

**Table 1:** Determination of specific parameters that tip the scale toward an anti-influenza state or exacerbation of influenza.

Group	Mouse strain	Time of AdE inoculation	Time of IFV challenge
1	Balb/c	Virus-free A195 buffer control on Day 38	PR8 on Day 39
2	Balb/c	AdE on Day 0	PR8 on Day 39
3	Balb/c	AdE on Day 38	PR8 on Day 39
4	Balb/c	AdE on Day 39	PR8 on Day 39 (co-inoculation with AdE)
5	Balb/c	AdE on Day 40	PR8 on Day 39
6	Balb/c	AdE on Days 0, 37, 38	PR8 on Day 39
7	Balb/c	AdE on Days 0, 37, 38, 40	PR8 on Day 39
8	Balb/c	Virus-free A195 buffer control on Day 38	CA04 on Day 39
9	Balb/c	AdE on Day 0	CA04 on Day 39
10	Balb/c	AdE on Day 38	CA04 on Day 39
11	Balb/c	AdE on Day 39	CA04 on Day 39 (co-inoculation with AdE)
12	Balb/c	AdE on Day 40	CA04 on Day 39
13	Balb/c	AdE on Days 0, 37, 38	CA04 on Day 39
14	Balb/c	AdE on Days 0, 37, 38, 40	CA04 on Day 39
15	C57BL/6	Virus-free A195 buffer control on Day 38	PR8 on Day 39
16	C57BL/6	AdE on Day 0	PR8 on Day 39
17	C57BL/6	AdE on Day 38	PR8 on Day 39
18	C57BL/6	AdE on Day 39	PR8 on Day 39 (co-inoculation with AdE)
19	C57BL/6	AdE on Day 40	PR8 on Day 39
20	C57BL/6	AdE on Days 0, 37, 38	PR8 on Day 39
21	C57BL/6	AdE on Days 0, 37, 38, 40	PR8 on Day 39
22	C57BL/6	Virus-free A195 buffer control on Day 38	CA04 on Day 39
23	C57BL/6	AdE on Day 0	CA04 on Day 39
24	C57BL/6	AdE on Day 38	CA04 on Day 39
25	C57BL/6	AdE on Day 39	CA04 on Day 39 (co-inoculation with AdE)
26	C57BL/6	AdE on Day 40	CA04 on Day 39
27	C57BL/6	AdE on Days 0, 37, 38	CA04 on Day 39
28	C57BL/6	AdE on Days 0, 37, 38, 40	CA04 on Day 39

There will be 13 young (2–3 months old) female mice per group; survival rates will be determined and body weights will be taken on a daily basis for 20 days post-IFV challenge; body-weight loss of > = 30% will be taken as the disease endpoint; A195 buffer, AdE, PR8, CA04 will be prepared as described [1] except AdE will be mass-produced in human 293 cells; statistical analysis will be performed as that described in Fig 1 legend. Three mice in each group will be euthanized 3 days post-IFV challenge, with their lungs resected for determining IFV titers by plaque assay in MDCK cells in conjunction with lung histopathology assay as described [1].

If our preliminary data should be reproducible and confirmed during these Stage I studies, we will establish a unique animal model that could enable the characterization of specific innate immunity signatures for predicting susceptibility or resistance to IFV infection. To test our driving hypothesis that AdE activates specific arms of innate immunity that impede IFV growth in the respiratory tract [5], we will analyze the lung during Stage II studies in search of biomarkers that may represent susceptibility or resistance to IFV infection. Lung samples will be collected at different time points from control, AdE-, IFV-, and AdE/IFV-infected mice; labeled antibodies against specific biomarkers (Table 2) will be used to analyze the panel of immune cells within the lung at varying time points. We will perform flowcytometric analysis of single cell suspensions of the lung to reveal local immunological changes at the portal whereby airborne viruses invade; activation of innate immune cell populations will be measured using CD69 as a marker of activation as described [6]; the % of activated natural killer (NK) cells (NK1.1^+^/CD3^-^), NKT cells (NK1.1^+^/CD3^+^), B cells (CD19^+^/CD3^-^), CD4^+^ T cells (CD3^+^/CD4^+^), CD8^+^ cytotoxic T cells (CD3^+^/CD8^+^), γδT cells (CD3^+^/TCR^+^), macrophages and dendritic cells (CD11b^+^/CD11c^+^) will be quantitatively determined by flowcytometry. Lung sections will be stained with labeled antibodies (Table 2) as described [7] for visual identification of varying classes of immune cells. Adaptive immune responses against AdE will be characterized by neutralization antibody assay as described [4] except human 293 cells will be infected. In addition, we will perform multiplex cytokine/chemokine profiling of lung lavages by assaying granulocyte macrophage colony-stimulating factor (GM-CSF), interferon (IFN)-γ, interleukin (IL)-1α, IL-2, IL-4, IL-6, IL-10, monocyte chemoattractant protein 1 (MCP-1), macrophage inflammatory protein 1β (MIP-1β), and tumor necrosis factor α (TNF-α) via Luminex technology as described [6]. These complementary shotgun approaches may collectively identify a specific innate immunity signature that represents susceptibility to IFV infection, as well as another distinct signature associated with resistance to IFV infection.

**Table 2:** Antibodies for characterizing immune cells that infiltrate into the lung as a response to pulmonary infection by AdE and/or IFV.

Labeled antibody	Mouse immune cells to be stained by the antibody
Anti-mouse CD3	All T cells
Anti-mouse CD4	CD4^+^ T cells
Anti-mouse CD8	CD8^+^ T cells
Anti-mouse δTCR	γδT cells
Anti-mouse NK1.1	NK and NKT cells
Anti-mouse CD19	B cells
Anti-mouse CD11b	Macrophages, dendritic cells, neutrophils, eosinophils
Anti-mouse CD11c	Macrophages and dendritic cells
Anti-mouse CD69	Marker for activation of innate immunity

We will characterize the panel of immune cells within the lung with a variety of labeled antibodies before and after intranasal instillation of AdE and/or IFV particles. Additional antibodies may be added to the list for expanding the scope as described [8].

In Stage II studies, we will also test the hypothesis by mitigating the hypothetical pro-IFV response induced by IFV infection with zanamivir (Relenza) and/or a variety of anti-inflammatory agents in an attempt to reset innate immunity within the airway post-IFV infection for allowing AdE to induce an anti-influenza state in a post-exposure-therapy manner. We will screen a variety of anti-inflammatory agents including aspirin [9], rapamycin [10], dexamethasone [11], etc. in a shotgun manner by intranasal instillation of an anti-inflammatory agent with or without zanamivir in conjunction with AdE administration at varying time points post-IFV challenge, and subsequently determine the agents’ effects on influenza by taking daily body weights and determining survival rates of IFV-infected mice.

## Ethics statement

Three biosafety protocols pertaining to coronavirus and IFV research have been approved by the University of North Texas Health Science Center (UNTHSC) Institutional Biosafety Committee (IBC) during 2019–2020; one animal-use-protocol application pertaining to these studies was approved by the University of North Texas Health Science Center Institutional Animal Care and Use Committee (UNTHSC IACUC) on October 8, 2020, with the approval number IACUC-2020-0022. The proposed mouse studies will be performed at the UNTHSC animal facility.

## Budget justification

### 1. Cell culture supplies

Mass production of AdE, PR8, and CA04 is fundamental to this project. The planned experiments in this project require propagation of human 293 cells for AdE production; and propagation of MDCK cells for PR8 and CA04 production. We are budgeting $4,000 for cell culture supplies.

### 2. Laboratory supplies and reagents

This budget includes HiTrap Capto Q columns for purification of viral particles, pipette tips, glassware, plasticware, cryovials and boxes, etc. We are asking for $1,000.

### 3. Animal costs

Animal studies will be performed within the animal facility at UNTHSC. Each inbred mouse costs approximately $25; and we will need approximately 400 mice with 5 mice per cage; mice will be maintained for approximately 30–70 days (a subset of mice is expected to be killed by influenza before the experiment is terminated). We are budgeting $20,000 for animal work (purchase and maintenance).

## Regulatory approval and support of the proposed work from University of North Texas Health Science Center

VaxDome was founded under the Commercialization Program at UNTHSC during 2019. UNTHSC has committed supporting VaxDome’s work on campus for 10 years as shown by the following documents that have been effective since 2019.

1. UNTprogram19: UNTHSC Acceleration Lab Program Participation Agreement
2. UNTprogram19amend: Amendment No. 1 to Acceleration Lab Program Participation Agreement
3. UNTfacility19: UNTHSC Facility Use Agreement
4. UNTfacility19amend: Amendment No. 1 to Facility Use Agreement
5. UNTfluIBC19: UNTHSC IBC approval of VaxDome’s protocol entitled “Innate-adaptive immunity duo vaccine against influenza”
6. UNTpaIBC20: UNTHSC IBC approval of VaxDome’s protocol entitled “Protective antigen as an innate immunity modulator”
7. UNTcvinfIBC20: UNTHSC IBC approval of VaxDome’s protocol entitled “BCG- and Ad-induced pathogen-agnostic protective effects against mouse coronavirus and/or mouse-adapted influenza virus”
8. UNTanimal19: UNTHSC Department of Laboratory Animal Medicine (DLAM) Services and Facility Use Agreement

## Supporting information

S1 File (**Table A**) Data set for determining survival rates post-CA04 challenge. (**Table B**) Data set fosr determining body weight loss post-CA04 challenge.
(DOCX)
