# Supplementary material for: Paradoxical modulation of influenza by intranasal administration of non-replicating adenovirus particles
Source: PLoS One. 2020 Nov 12;15(11):e0241266. doi: 10.1371/journal.pone.0241266 (PMC7660471; doi:10.1371/journal.pone.0241266)
Supplement: S1 File — (Table A) Data set for determining survival rates post-CA04 challenge. (Table B) Data set fosr determining body weight loss post-CA04 challenge. (DOCX) [file pone.0241266.s001.docx]

**Supplementary information**

**Table A. Data set for determining survival rates of mice post-CA04 challenge**

| **Day** | **Buffer-1 (10)** | **AdE-39 (10)** | **AdE-1**  **(10)** | **AdHA-1 (9)** | **Ad-39-2-1 (10)** | **AdE+1 (10)** |
| --- | --- | --- | --- | --- | --- | --- |
| 0 | 100 | 100 | 100 | 100 | 100 | 100 |
| 2 | 100 | 100 | 100 | 100 | 100 | 100 |
| 4 | 100 | 100 | 100 | 100 | 100 | 50 |
| 6 | 100 | 70 | 90 | 89 | 100 | 50 |
| 8 | 90 | 40 | 80 | 78 | 100 | 30 |
| 10 | 60 | 30 | 70 | 67 | 100 | 30 |
| 12 | 60 | 10 | 70 | 67 | 100 | 30 |
| 14 | 60 | 10 | 70 | 55 | 100 | 20 |
| 16 | 60 | 10 | 70 | 55 | 100 | 20 |
| 18 | 60 | 10 | 70 | 55 | 100 | 20 |
| 20 | 60 | 10 | 70 | 55 | 100 | 20 |

**Table B. Data set for determining body weight loss post-CA04 challenge**

| **Day** | **Buffer-1 (10)** | **AdE-39 (10)** | **AdE-1**  **(10)** | **AdHA-1 (9)** | **Ad-39-2-1 (10)** | **AdE+1 (10)** |
| --- | --- | --- | --- | --- | --- | --- |
| 0 | 100 | 100 | 100 | 100 | 100 | 100 |
| 2 | 96 | 90 | 97 | 95 | 101 | 91 |
| 4 | 90 | 80 | 95 | 87 | 102 | 85 |
| 6 | 83 | 74 | 92 | 82 | 103 | 77 |

AdE and AdCA04HA1 particles as well as CA04 IFV particles were inoculated into young (2-3 months old) female C57BL/6 mice by intranasal instillation at different time points. Mice were monitored daily for survival with body weights recorded every other day post-challenge. (**Table A**) Data set for determining survival rates post-CA04 challenge; % survival was determined by taking the number of mice on Day 0 as 100%. (**Table B**) Body weight loss post-CA04 challenge; post-challenge body weights are presented as mean % body weight by taking the body weight of individual mice on Day 0 as 100%. Buffer-1 (negative control group inoculated with virus-free A195 buffer only), A195 buffer was instilled into the nostril one day prior to CA04 challenge; AdE-39, AdE particles were instilled into the nostril 39 days prior to CA04 challenge; AdE-1, AdE particles were instilled into the nostril 1 day prior to CA04 challenge; AdHA-1, AdCA04HA1 particles were instilled into the nostril 1 day prior to CA04 challenge; Ad39-2-1, AdE particles were instilled into the nostril 39 and 2 days prior to CA04 challenge, followed by instillation of AdCA04HA1 particles 1 day prior to CA04 challenge; AdE+1, AdE particles were instilled into the nostril 1 day after CA04 challenge; parentheses represent the number of animals in each group. AdE and AdCA04HA1 particles were instilled into the nostril at a dose of 1.6X10^8^ infectious units (ifu) in a volume of 0.05 ml per mouse; CA04 particles were instilled into the nostril at a dose of 8X10^3^ plaque-forming units (pfu) in a volume of 0.05 ml per mouse, which was equivalent to approximately 1xLD_50_ in this experiment.
